# Regulation of Cardiac Mast Cell Maturation and Function by the Neurokinin-1 Receptor in the Fibrotic Heart

**DOI:** 10.1038/s41598-019-47369-0

**Published:** 2019-07-29

**Authors:** Alexander Widiapradja, Edward J. Manteufel, Heather M. Dehlin, James Pena, Paul H. Goldspink, Amit Sharma, Lauren L. Kolb, John D. Imig, Joseph S. Janicki, Bao Lu, Scott P. Levick

**Affiliations:** 1 Kolling Institute of Medical Research, Royal North Shore Hospital, St Leonards, NSW 2065 Australia; 2 Faculty of Medicine and Health, The University of Sydney, Camperdown, NSW 2006 Australia; 3 Department of Pharmacology and Toxicology, Medical College of Wisconsin, Milwaukee, WI 53226 USA; 4 Department of Physiology, Medical College of Wisconsin, Milwaukee, WI 53226 USA; 5 Cell Biology and Anatomy, University of South Carolina School of Medicine, Columbia, SC 29208 USA; 6 Division of Respiratory Diseases, Boston Children’s Hospital, Harvard Medical School, Boston, MA 02115 USA

**Keywords:** Cardiac hypertrophy, Heart failure

## Abstract

Cardiac fibrosis is an underlying cause of diastolic dysfunction, contributing to heart failure. Substance P (SP) activation of the neurokinin-1 receptor (NK-1R) contributes to cardiac fibrosis in hypertension. However, based on *in vitro* experiments, this does not appear to be via direct activation of cardiac fibroblasts. While numerous cells could mediate the fibrotic effects of SP, herein, we investigate mast cells (MC) as a mechanism mediating the fibrotic actions of SP, since MCs are known to play a role in cardiac fibrosis and respond to SP. Spontaneously hypertensive rats (SHR) were treated with the NK-1R antagonist L732138 (5 mg/kg/d) from 8 to 12 weeks of age. L732138 prevented increased MC maturation of resident immature MCs. NK-1R blockade also prevented increased cardiac MC maturation in angiotensin II-infused mice. MC-deficient mice were used to test the importance of MC NK-1Rs to MC activation. MC-deficient mice administered angiotensin II did not develop fibrosis; MC-deficient mice reconstituted with MCs did develop fibrosis. MC-deficient mice reconstituted with MCs lacking the NK-1R also developed fibrosis, indicating that NK-1Rs are not required for MC activation in this setting. In conclusion, the NK-1R causes MC maturation, however, other stimuli are required to activate MCs to cause fibrosis.

## Introduction

During the progression to heart failure, the left ventricle (LV) enlarges due to hypertrophy of cardiomyocytes. Accompanying this are changes to components of the extracellular matrix (ECM). Most important of these ECM alterations is the accumulation of excess fibrillar collagen, contributing to cardiac fibrosis^1,2^. The functional manifestation of cardiac fibrosis includes increased myocardial stiffness and diastolic dysfunction, which contribute to impaired LV filling and ultimately heart failure. With the increasing incidence of heart failure with preserved ejection fraction, of which fibrosis is a major risk factor, there has been renewed interest in the mechanisms underlying cardiac fibrosis in the hope of identifying new therapeutic targets. To date, there are currently no specific treatments for cardiac fibrosis or heart failure with preserved ejection fraction.

We recently reported that the neuropeptide substance P (SP) and its cognate receptor, the neurokinin-1 receptor (NK-1R), are critical to the development of cardiac fibrosis in the hypertensive rat heart^3^. Interestingly though, we found that while cardiac fibroblasts possess the NK-1R, they do not convert to myofibroblasts or synthesize excess amounts of ECM proteins in response to SP^3,4^. Thus, other indirect pathways must be involved. To this end, SP has the ability to act as a cardiac mast cell (MC) secretagogue *in vitro* via the NK-1R^5–7^. This is of significance because cardiac MCs play an important role in fibrosis in the heart^8^. For example, MC tryptase acts via protease activated receptor-2 to initiate ERK signaling resulting in cardiac fibroblast conversion to the myofibroblast phenotype, excess collagen synthesis, and cardiac fibrosis^9,10^. Additionally, MC chymase promotes cardiac fibrosis via activation of transforming growth factor- β1 (TGF-β1), as well as cleavage of angiotensin I to active angiotensin II^11,12^. However, whether MCs mediate the pro-fibrotic actions of SP and the NK-1R has not been examined.

Using a combination of *in vitro* MC cultures and *in vivo* approaches including MC-deficient mice reconstituted with MCs lacking the NK-1R, we determined that the NK-1R regulates MC maturation in the pressure overloaded heart by up-regulating MC survival signals including stem cell factor (SCF). However, MC-specific NK-1Rs are not required for MC activation in the fibrotic heart. We also report that the NK-1R regulates myofibroblast numbers in the hypertensive heart.

## Methods

### Animals

Experiments were performed using male rodents; these included spontaneously hypertensive rats (SHR) and the normotensive control wistar kyoto (WKY) rat, as well as wild type, *Tac1*^−/−^, *Nk-1r*^−/−^, tumor necrosis factor receptor I *(TnfrI*^−/−^), and B6.Cg-*Kit*^*W-sh*^/HNihrJaeBsmGlliJ (*Kit*^*w-sh/w-sh*^) MC-deficient mice. All mice were on the C57BL/6 background. Wild type, *Tac1*^−/−^*, TnfrI*^−/−^, and *Kit*^*w-sh/w-sh*^ mice were purchased from Jackson Laboratories. *Nk-1r*^−/−^ were previously developed at Harvard University. All rodents were housed under standard environmental conditions and maintained on standard commercial rat/mouse chow and tap water *ad libitum*. All animals were anesthetized by inhaled isoflurane prior to euthanasia. Proper analgesia for euthanasia was evaluated by palpebral reflex, toe pinch reflex, and corneal reflex. At the experimental endpoint, euthanasia was accomplished by removal of the heart. These studies conformed to the principles of the National Institutes of Health *Guide for the Care and Use of Laboratory Animals*, and the protocols were approved by the Institutional Animal Care and Use Committees.

### Experimental groups

The SHR model of hypertension was chosen to initially assess the action of the NK-1R on cardiac MCs since we had previously shown that blockade of the NK-1R with L732138 (5 mg/kg/day) prevented cardiac fibrosis in this model^3^. SHR and WKY rats were euthanized at 8, 12, and 16 weeks of age (n = 4–6/time-point). A separate group of SHR (n = 8) were treated with the NK-1R antagonist, L732138 (5 mg/kg/day) by oral gavage from 8 to 12 weeks of age. In order to examine the role of SP in cardiac fibrosis in mice, 8-week-old wild type (n = 7) and *Tac1*^−/−^ (n = 7) mice were treated with angiotensin II (1500 ng/kg/min, osmotic mini-pump) for 7 days. *Tac1* is the gene that encodes SP. Wild type saline (n = 7) and *Tac1*^−/−^ saline mice (n = 6) served as controls. To investigate the involvement of the NK-1R in regulating MC numbers in mice, 8-week-old wild type mice were treated with L732138 (5 mg/kg/d) for 1 day. To determine the contribution of MC-specific NK-1Rs to cardiac fibrosis, 14 week old *Kit*^*w-sh/w-sh*^ mice were divided into 3 groups: 1) *Kit*^*w-sh/w-sh*^ mice + angiotensin II (1500 ng/kg/min, osmotic mini-pump, n = 7) for 7 days; 2) *Kit*^*w-sh/w-sh*^ mice receiving wild type bone marrow-derived mast cells (BMMCs) + angiotensin II (n = 7); and 3) *Kit*^*w-sh/w-sh*^ mice receiving *Nk-1r*^−/−^ BMMCs + angiotensin II (n = 6). To examine the role of MC-specific TNFRI to MC activation in cardiac fibrosis, *Kit*^*w-sh/w-sh*^ mice received *TnfrI*^−/−^ BMMCs prior to angiotensin II infusion (n = 8). Systolic blood pressure was measured by tail cuff method. At the time of euthanasia, the atria and great vessels were dissected away, and the LV and right ventricle (RV) separated. The apical portion of the LV was snap frozen in liquid nitrogen and stored at −80 °C, while the mid-papillary portion was fixed in zinc formalin for histology analysis. Additional methods are provided in Supplementary Information.

## Results

### The NK-1R regulates cardiac MC maturation in the hypertensive rat heart

**Figure 1:**
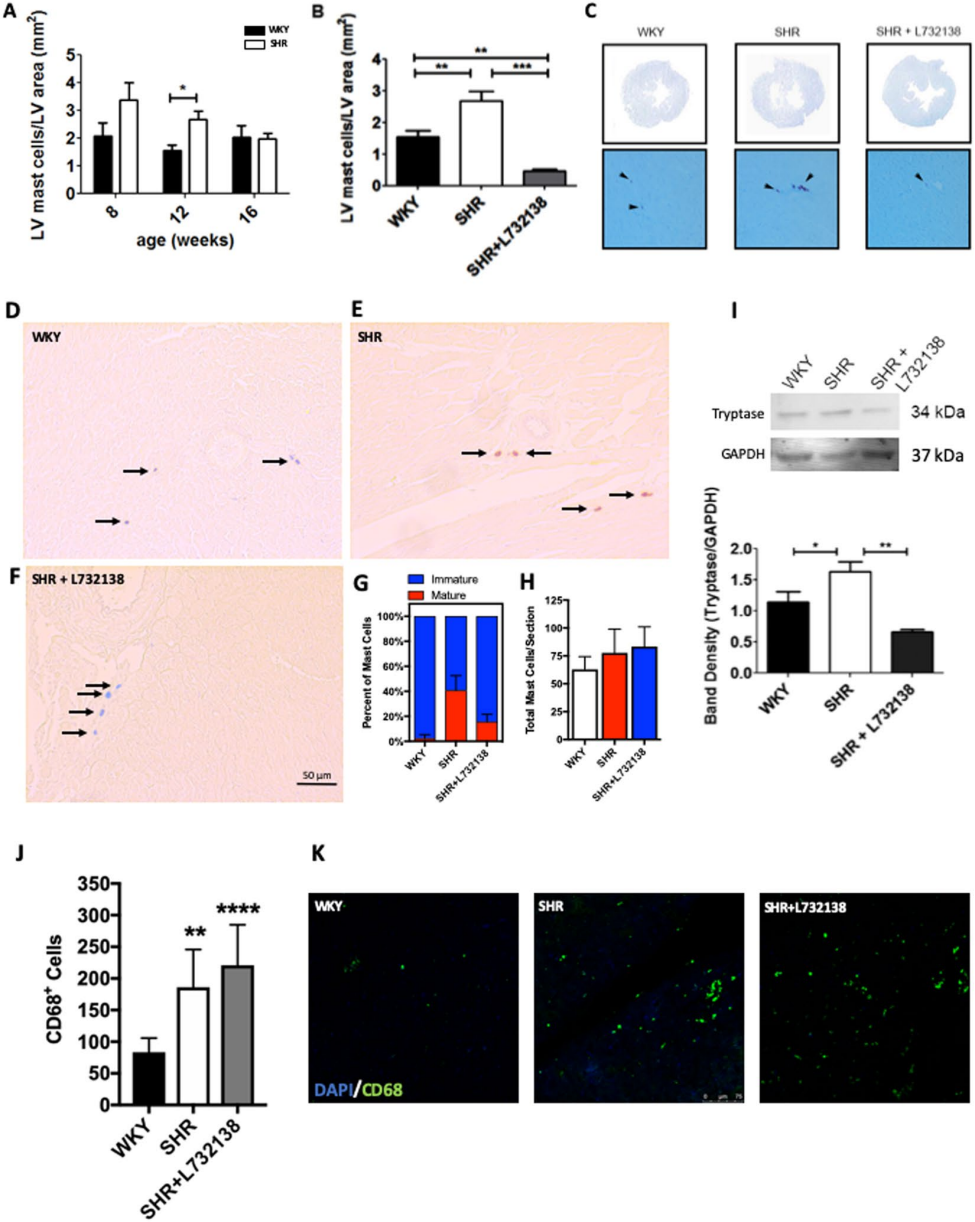
The NK-1R regulates MC maturation in the hypertensive heart. (**A)** Cardiac MC density as determined using toluidine blue staining in SHR and WKY LV from 8, 12, and 16 weeks of age (n = 4–6); **(B**,**C)** L732138 (5 mg/kg/d) treatment from 8 to 12 weeks of age prevented the increase in cardiac MC density in SHR (n = 6–8). All values are mean ± SEM; *P < 0.05, **P < 0.01, ***P < 0.001. **(D**–**F)** Representative images and **(G)** Quantification of the percentage of immature and mature cardiac MCs as determined using alcian blue/safranin staining (Blue stained MCs = immature, brick red stained MCs = mature); Arrows indicate MCs. **(H)** Total number of cardiac MCs (immature + mature). All values are mean ± SD (n = 8/group); P < 0.0001 for WKY vs SHR, P < 0.05 for SHR vs SHR + L732138, P < 0.001 for WKY vs SHR + L732138. **(I)** Representative western blot and band quantification for tryptase and GAPDH in LV from 12 wk old WKY, SHR, and SHR treated with L732138. Tryptase was normalized to GAPDH. Values are mean ± SEM; *P < 0.05, **p < 0.01. **(J)** Quantification of CD68^+^ macrophages in LV sections from WKY, SHR, and SHR treated with L732138 (n = 8/group). Values are mean ± SD; **P < 0.01, ****P < 0.0001 vs WKY; **(K)** Representative images of CD68^+^ macrophages (Green). Blue = DAPI nuclear labeling.

We established the temporal MC response in SHR during the development of fibrosis by determining cardiac MC density at 8, 12, and 16 weeks of age using toluidine blue stain. MC density was significantly higher in 12-week old SHR compared to WKY, before returning to normal by 16 weeks of age (Fig. 1A). This corresponds with the period of initiation of cardiac fibrosis in this model. Subsequently, we treated SHR with the NK-1R antagonist L732138 from 8 to 12 weeks of age and observed that the increase in MC density was prevented (Fig. 1B,C), and in fact fell below control levels. Toluidine blue stains MCs by labeling heparin sulfate within MC granules, meaning that the identified MCs are mature MCs since immature MCs do not contain heparin sulfate^13,14^. To further confirm maturation and to investigate immature MCs, we also stained LV sections with alcian blue-safranin, where alcian blue stains immature MCs and safranin stains mature MCs. We found a significant increase in the percentage of mature MCs (safranin^+^) and a corresponding decrease in immature MCs (alcian blue^+^/safranin^−^) in SHR LVs (Fig. 1D–G). Overall number of MCs did not change (immature + mature, Fig. 1H), indicating that there are not more MCs in the hypertensive heart, just a shift to more mature MCs. This increased maturation, but not the overall number of MCs, was significantly reduced by NK-1R blockade with L732138 (Fig. 1D–H). Consistent with increased MC maturation, there was an increase in tryptase in the SHR heart compared to the WKY, which was normalized by L732138 (Fig. 1I). We also examined the effect of NK-1R blockade on cardiac macrophage numbers, identified as CD68^+^ cells. There was a significant increase in CD68^+^ cells in the SHR hearts, which was unaffected by NK-1R blockade with L732138 (Fig. 1J,K).

### The NK-1R up-regulates SCF as a mechanism underlying MC maturation, but does not alter MC c-kit levels, apoptosis or proliferation

**Figure 2:**
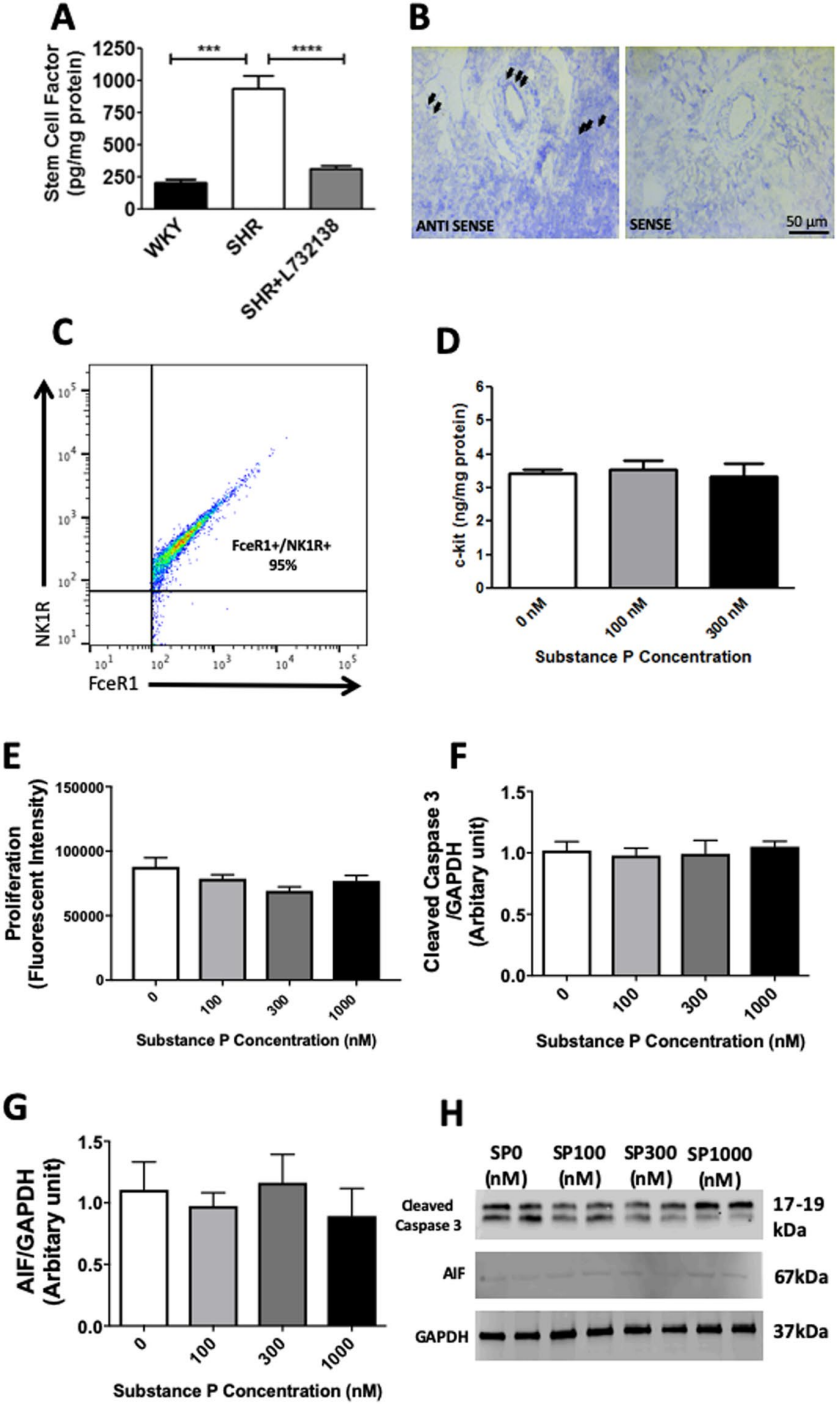
The NK-1R increases SCF production, but does not alter MC proliferation or apoptosis. **(A)** Stem cell factor levels for SHR, WKY, and SHR treated with the NK-1R antagonist L732138 (5 mg/kg/d). All values are mean ± SEM; n = 6–8; ***P < 0.001, ****P < 0.0001; **(B)** *In situ* hybridization identifying cells expressing SCF (antisense) in the left ventricle. Endothelial cells and cardiomyocytes labeled positive for stem cell factor (black arrows). Incubation with sense probes served as the negative control; **(C)** Flow cytometry analysis of BMMCs (FcεRI^+^ cells) indicating that 95% of BMMCs possess the NK-1R; **(D)** c-kit protein levels on BMMCs as determined by ELISA; **(E)** BMMC proliferation assay (n = 12); Quantification of cleaved caspase-3 **(F)** and apoptosis inducing factor (AIF) **(G)**; Representative western blots for cleaved caspase-3 and AIF **(H)** from BMMCs treated with SP (n = 6). All values are mean ± SEM. Gel images in (**D**) are cropped from three separate blots. Images of full-length blots are included in Fig. S7.

Having shown that the NK-1R regulates cardiac MC maturation, we then sought to determine the mechanisms involved. SCF is essential for MC survival, proliferation and maturation^15–18^ and was significantly up-regulated in SHR LV at 12 weeks of age (Fig. 2A). NK-1R antagonism with L732138 prevented this increase. *In situ* hybridization identified that SCF was produced by endothelial cells and cardiomyocytes (Fig. 2B), as well as interstitial cells (likely fibroblasts). To investigate NK-1R regulation of MC levels of c-kit, the receptor for SCF, we utilized an *in vitro* model of BMMCs as a surrogate for cardiac MCs. Flow cytometry determined that approximately 95% of BMMCs (FcεRI^+^ cells) possessed the NK-1R (Figs 2C, S1). Treatment with SP did not increase c-kit on BMMCs (Fig. 2D).

In addition to maturation, SCF is also capable of inducing MC proliferation. We labelled sections of SHR hearts with Ki67 as a marker of cellular proliferation, together with alcian blue as a marker of MCs. Despite numerous cells being independently Ki67^+^ or alcian blue^+^, we found only one cell that was both Ki67^+^ and alcian blue^+^ in LV sections from six individual SHR hearts examined (data not shown). We confirmed this lack of a proliferative response *in vitro* where we found no increase in the proliferation of BMMCs treated with SP (Fig. 2E). Thus, we concluded that MC proliferation does not significantly contribute to the increase in MCs in the SHR heart. Reduced MC apoptosis could also lead to increased MC numbers. SP did not alter caspase-dependent or caspase-independent apoptosis of BMMCs as determined by cleaved caspase-3 and apoptosis inducing factor (AIF), respectively (Fig. 2F–H).

### SP does not cause MCs to release products to induce a pro-fibrotic phenotype in fibroblasts *in vitro*

**Figure 3:**
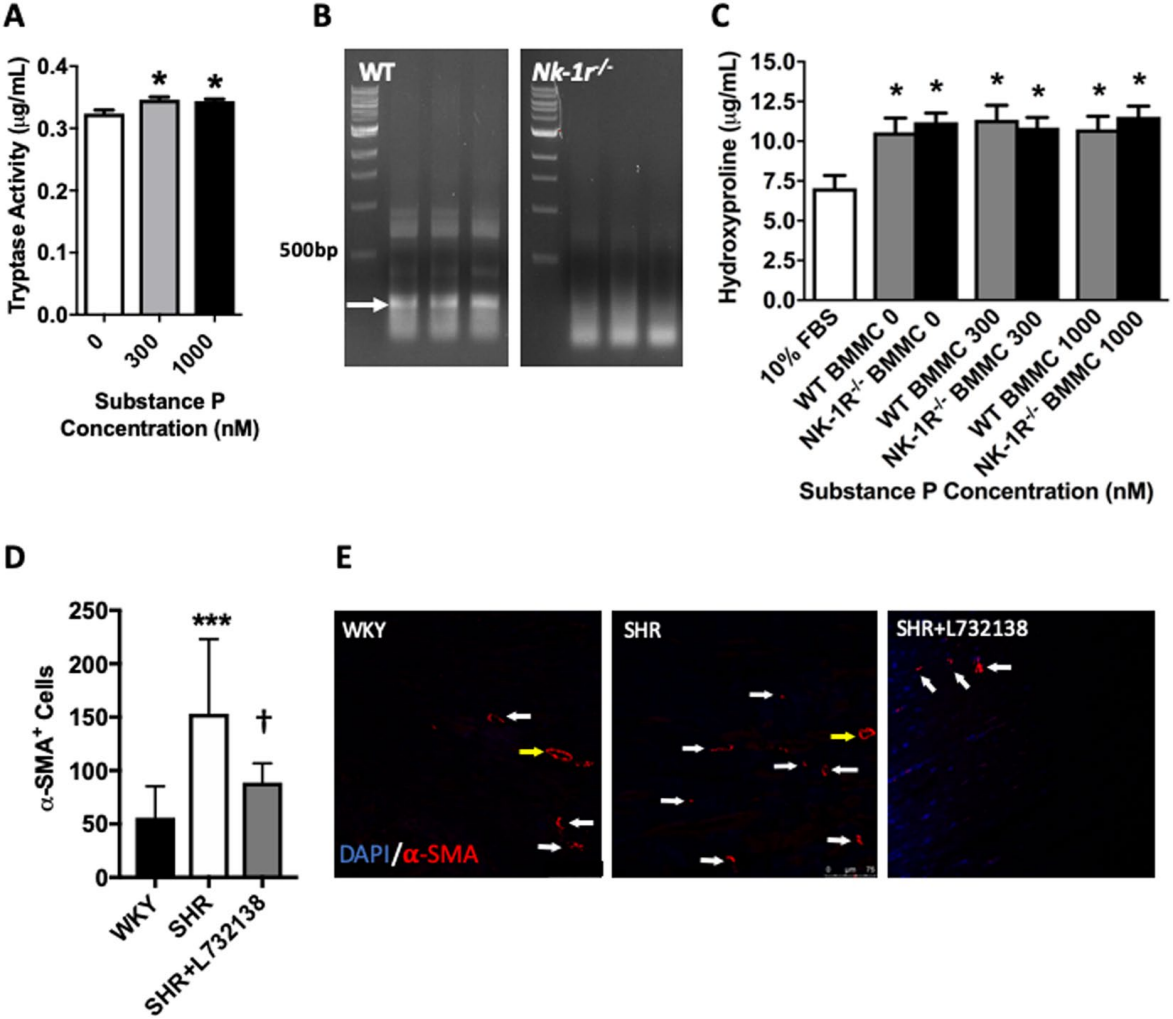
SP does not cause MCs to produce sufficient amounts of pro-fibrotic mediators to influence fibroblast function *in vitro*, but does increase myofibroblast number *in vivo*. (**A**) Tryptase activity in the media of BMMCs treated with SP (n = 4); **(B)** PCR showing disruption of the PCR product for the NK-R in BMMCs derived from *Nk-1r*^−/−^ mice; **(C)** Hydroxyproline production by 3T3 fibroblasts in response to conditioned media from SP treated BMMCs (n = 15). SP concentrations refer to the treatment of the BMMCs. 10% FBS served as a control. All values are mean ± SEM; *P < 0.05 vs SP 0 nM for (**A**) and vs 10% FBS for (**C**). (**D**) Quantification of α-smooth muscle actin (SMA)^+^ myofibroblasts in LV sections from WKY, SHR, and SHR treated with L732138 (n = 8/group). Values are mean ± SD; ***P < 0.001 vs WKY, ^†^p < 0.05 vs SHR; (**E**) Representative images of α-SMA^+^ myofibroblasts (red = α-SMA). Blue = DAPI nuclear labeling, yellow arrow = blood vessels, white arrow = myofibroblast.

We examined the ability of SP and the NK-1R to activate MCs *in vitro* using BMMCs. Using tryptase activity in the media as a marker of BMMC activation, we found that 24 hours treatment with SP at 300 nM and 1000 nM induced a very small, but statistically significant release of active tryptase (Fig. 3A). Accordingly, we collected conditioned media from wild type BMMCs and *Nk-1r*^−/−^ BMMCs (Fig. 3B), following treatment with SP (300 nM and 1000 nM) for 24 hours. We then incubated 3T3 fibroblasts with this BMMC conditioned media for a further 24 hours and measured the release of hydroxyproline by fibroblasts as a surrogate marker for collagen production. 3T3 fibroblasts increased the release of hydroxyproline in response to conditioned media from BMMCs, but did not synthesize additional hydroxyproline in response to conditioned media from BMMCs treated with SP (Fig. 3C). Consequently, deletion of the NK-1R in BMMCs did not alter hydroxyproline synthesis by 3T3 fibroblasts (Fig. 3C).

### NK-1R blockade regulates myofibroblast levels *in vivo*

SHR hearts had an increased number of myofibroblasts (interstitial α-smooth muscle actin^+^ cells) compared to the WKY controls (Fig. 3D,E). NK-1R blockade with L732138 in the SHR normalized the number of myofibroblasts (Fig. 3D,E).

### MC-Specific NK-1Rs are not involved in MC activation resulting in fibrosis

**Figure 4:**
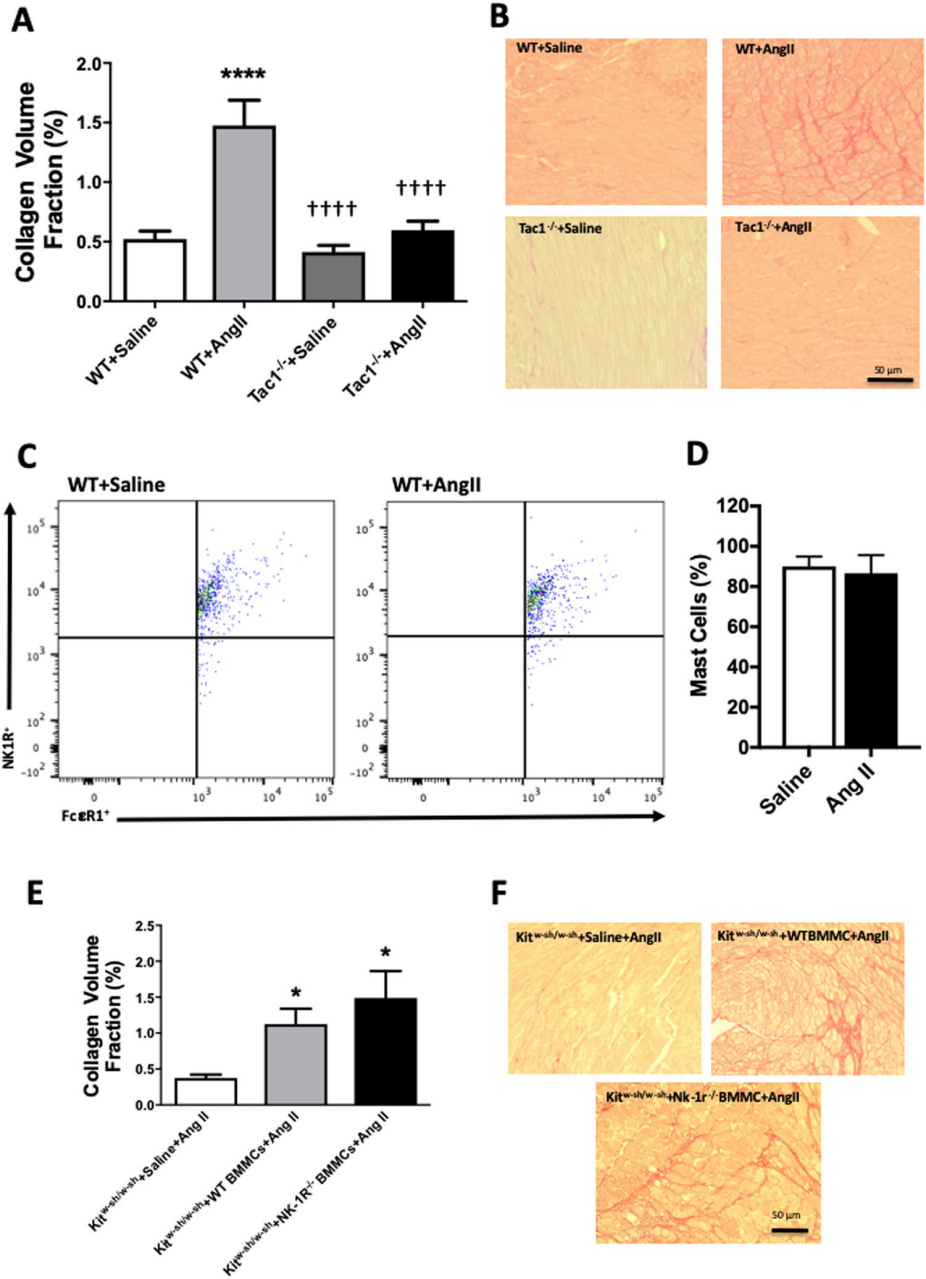
SP contributes to cardiac fibrosis, however, MC-specific NK-1Rs do not play a functional role in the activation of MCs and cardiac fibrosis *in vivo*. (**A)** Collagen volume fraction for WT saline (n = 7), WT angiotensin II (n = 7), *Tac1*^−/−^ saline (n = 6), and *Tac1*^−/−^ angiotensin II (n = 7) mice; **(B)** Corresponding representative picrosirious red images; **(C)** Representative flow cytometry scatter plots for WT saline and WT angiotensin II mice, indicating the percent of cardiac MCs that possess the NK-1R; **(D)** Quantitative flow cytometry analysis of the percentage of cardiac MCs possessing the NK-1R; **(E)** Collagen volume fraction for *Kit*^*w-sh/w-sh*^ mice receiving angiotensin II with no BMMCs (n = 7), *Kit*^*w-sh/w-sh*^ mice receiving wild type BMMCs and angiotensin II (n = 7), and *Kit*^*w-sh/w-sh*^ receiving *Nk-1r*^−/−^ BMMCs and angiotensin II (n = 6); and **(F)** corresponding representative picrosirius red images. All values are mean ± SEM; *P < 0.05, ****P < 0.0001 vs saline, ^††††^p < 0.0001 vs Ang II.

We then sought to examine the contribution of MC-specific NK-1Rs to cardiac fibrosis *in vivo* using *Kit*^*w-sh/w-sh*^ MC-deficient mice infused with angiotensin II. Firstly though, we confirmed that SP contributes to cardiac fibrosis in the angiotensin II mouse model. Wild type and *Tac1*^−/−^ mice were infused with either saline or angiotensin II for 7 days via osmotic mini-pumps. *Tac1* is the gene that encodes SP. Biometric data for these mice can be found in Table 1. Fibrosis was prevented in *Tac1*^−/−^ hearts (Fig. 4A,B). Next, we confirmed that mouse cardiac MCs possess the NK-1R. Flow cytometry analysis showed that 91% of cardiac MCs expressed the NK-1R, which was not altered by angiotensin II-infusion (Figs 4C,D, S2). We then confirmed that it was possible to reconstitute the hearts of *Kit*^*w-sh/w-sh*^ mice with BMMCs. Using Q-dot labeling of injected BMMCs, we tracked these MCs to the hearts of *Kit*^*w-sh/w-sh*^ mice 6 weeks after tail vein injection (Fig. S3). In order to address the issue of the importance of MC-specific NK-1Rs to MC activation and subsequent cardiac fibrosis, we reconstituted *Kit*^*w-sh/w-sh*^ mice with either wild type or *Nk-1r*^−/−^ BMMCs. Biometric data for these mice can be found in Table 2. Both groups reconstituted equal amounts of MCs (Fig. S4). Angiotensin II infusion for 7 days, did not cause cardiac fibrosis in control *Kit*^*w-sh/w-sh*^ mice that received saline instead of BMMCs (Fig. 4E,F). Conversely, *Kit*^*w-sh/w-sh*^ mice reconstituted with wild type BMMCs did develop cardiac fibrosis in response to angiotensin II (Fig. 4E,F). *Kit*^*w-sh/w-sh*^ mice that received MCs lacking the NK-1R also developed cardiac fibrosis (Fig. 4E,F).

**Table 1:** *Tac1*^−/−^ mice biometric data.

	BW (g)	LV (mg)	LV/BW (mg/g)	RV (mg)	RV/BW (mg/g)	SBP (mmHg)
WT + Saline (n = 7)	24.1 ± 0.6	74.4 ± 1.3	3.09 ± 0.11	25.0 ± 1.4	1.03 ± 0.05	100.1 ± 2.6
WT + ang II (n = 7)	21.6 ± 0.6*	68.7 ± 3.6	3.18 ± 0.14	18.5 ± 0.1*	0.85 ± 0.07	107.3 ± 1.2
*Tac1*^−/−^ + Saline (n = 6)	22.9 ± 0.81	75.9 ± 9.0	3.31 ± 0.37	23.7 ± 4.7	1.03 ± 0.20	106.1 ± 0.2
*Tac1*^−/−^ + ang II (n = 7)	23.2 ± 1.1	79.8 ± 10.7	3.44 ± 0.35	20.8 ± 3.3	0.90 ± 0.16	121.2 ± 7.7*^,†,‡^

WT = wild type, BW = body weight, LV = left ventricle, RV = right ventricle, SBP = systolic blood pressure. Mean ± SD, *p < 0.05 vs WT Saline, ^†^p < 0.05 vs WT ang II, ^‡^p < 0.05 vs *Tac1*^−/−^ Saline.

**Table 2:** *Kit*^*w-sh/w-sh*^ mice biometric data.

	BW (g)	LV (mg)	LV/BW (mg/g)	RV (mg)	RV/BW (mg/g)	SBP (mmHg)
*Kit*^*w-sh/w-sh*^ + Saline + ang II (n = 7)	24.8 ± 1.1	94.1 ± 9.0	3.79 ± 0.24	24.0 ± 3.3	0.97 ± 0.15	106.8 ± 9.9
*Kit*^*w-sh/w-sh*^ + WT BMMC + ang II (n = 7)	24.8 ± 2.2	94.7 ± 7.0	3.84 ± 0.30	23.7 ± 2.6	0.96 ± 0.15	123.2 ± 10.7
*Kit*^*w-sh/w-sh*^ + NK-1R^−/−^BMMC + ang II (n = 6)	26.3 ± 4.7	104.8 ± 15.9	4.02 ± 0.49	26.7 ± 5.7	1.01 ± 0.09	119.7 ± 9.6

BMMC = bone marrow derived mast cell, WT = wild type, BW = body weight, LV = left ventricle, RV = right ventricle, SBP = systolic blood pressure. Mean ± SD.

### TNF-α does not contribute directly to MC activation in the fibrotic heart

**Figure 5:**
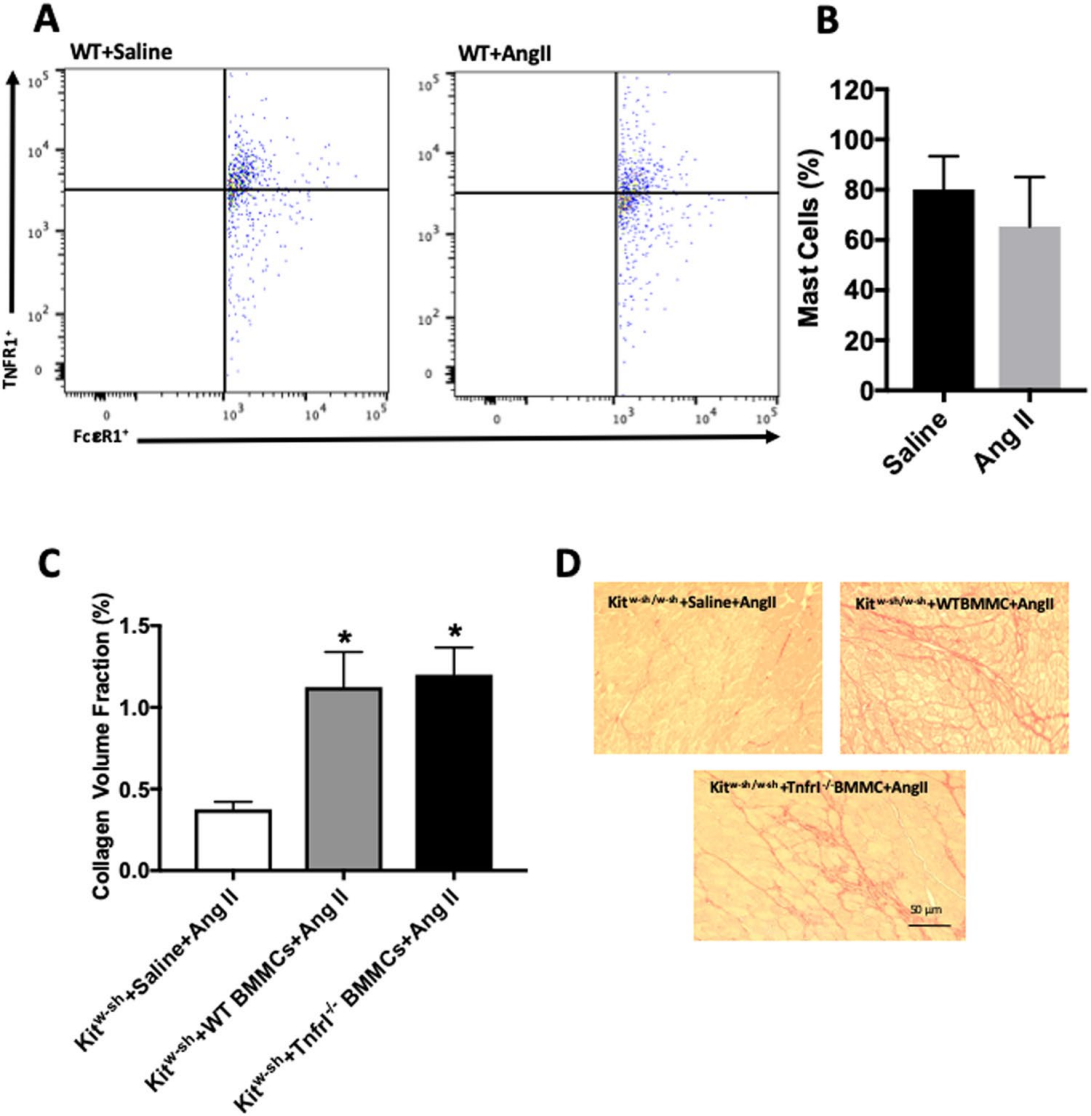
MC-specific TNFRI does not play a functional role in the activation of MCs and cardiac fibrosis *in vivo*. (**A**) Representative flow cytometry scatter plots indicating the percentage of MCs that possess TNFRI, and **(B)** quantitative flow cytometry analysis of the percentage of MCs possessing TNFRI in left ventricles from wild type mice receiving saline or angiotensin II; **(C)** Collagen volume fraction for *Kit*^*w-sh/w-sh*^ mice receiving angiotensin II with no BMMCs (n = 7), *Kit*^*w-sh/w-sh*^ mice receiving wild type BMMCs and angiotensin II (n = 7), and *Kit*^*w-sh/w-sh*^ receiving *TnfrI*^−/−^ BMMCs and angiotensin II (n = 8); and **(D)** corresponding representative picrosirius red images. All values are mean ± SEM. *P < 0.05 vs *Kit*^*w-sh/w-sh*^ mice receiving angiotensin II with no BMMCs.

Having ruled out the NK-1R as directly activating cardiac MCs to cause fibrosis, we turned our attention to TNF-α since cardiac fibrosis in TNF-α overexpressing mice is abolished when these mice are crossed with MC-deficient mice, implicating TNF-α in MC activation in the fibrotic heart^19^. However, it was unclear if this was a direct effect. Further, SP is known to regulate TNF-α in the rodent heart^5^, thus, TNF-α could serve as an intermediate between SP and MCs. We identified that 80% of mouse cardiac MCs expressed TNFRI and that this did not differ significantly with angiotensin II (Figs 5A,B, S5). We then reconstituted *Kit*^*w-sh/w-sh*^ mice with BMMCs deficient in TNFRI (Fig. S4). Angiotensin II infusion for 7 days caused fibrosis in *Kit*^*w-sh/w-sh*^ mice reconstituted with wild type BMMCs (Fig. 5C,D). *Kit*^*w-sh/w-sh*^ mice that received *TnfrI*^−/−^ BMMCs also developed cardiac fibrosis (Fig. 5C,D).

### NK-1R blockade prevents increased MC maturation in angiotensin II-infused mice

We also sought to assess whether the same regulation of MC maturation and SCF that occurred by SP and the NK-1R in the SHR heart also occurred in the angiotensin II mouse heart. Mouse cardiac MCs do not stain well with alcian blue-safranin, so instead we used avidin, which also labels mature MCs. Cardiac MC numbers tended to be elevated in wild type mouse hearts following 7 days of angiotensin II infusion (Fig. S6A). While MC numbers tended to be decreased in *Tac1*^−/−^ mice, there was large variability in the number of MCs in all groups. We wondered whether this could be an artifact of germ line deletion of SP in the *Tac1*^−/−^ mice. Accordingly, we assessed MC number in wild type mice infused with angiotensin II for 1 day and treated with the NK-1R antagonist L732138, as was the case for the SHR. The number of MCs was increased in response to angiotensin II, with this increase prevented by NK-1R blockade (Fig. S6B). We then assessed cardiac SCF levels following angiotensin II infusion in mice and found no increase in SCF (Fig. S6C).

## Discussion

Previously we reported that the NK-1R mediates cardiac fibrosis in SHR hearts^3^. However, *in vitro* data suggest that this does not involve direct activation of cardiac fibroblasts even though these cells possess the NK-1R. Thus, intermediate steps must be involved. Studies from our laboratory and others have provided causal evidence that MCs also contribute to cardiac fibrosis^9,10,12,20^. Therefore, we examined whether neuro-immune mechanisms contribute to cardiac fibrosis by investigating whether MCs mediate the pro-fibrotic actions of SP and the NK-1R.

Initially we used the SHR model to investigate cardiac MC number since this was the model in which we had previously established the contribution of the NK-1R to cardiac fibrosis^3^. Using toluidine blue, we found that the NK-1R did regulate the increase in MC density in the SHR heart. Toluidine blue stains mature, but not immature MCs since it labels heparin sulfate, which is present in the granules of mature MCs, but not immature MCs^13,14^. Therefore, the observed increase in MCs was an increase in mature MCs. To confirm this MC maturation and to determine effects on immature MCs, we then stained LV sections with alcian blue-safranin, which identifies mature (safranin) as well as immature (alcian blue) MCs. This confirmed NK-1R-driven MC maturation in the SHR heart, which was due to maturation of resident immature MCs rather than an influx of new MCs that then mature, since total MC numbers (mature + immature cells) were unchanged. In a model of volume overload, we previously reported that NK-1R antagonism prevented increased MC density, which is also known to be due to MC maturation in that model^5,21^. Thus, MC maturation may be a conserved response in the heart to rapidly increase the number of effector MCs, regardless of the type of overload.

We also examined MC proliferation, however, this does not appear to occur at any meaningful level in the heart since we were only able to identify one proliferating MC in LV sections from six individual SHR hearts. *In vitro* treatment of BMMCs also confirmed no proliferative effects of SP. An additional possibility underlying the increase in MC density could be that the NK-1R reduced MC apoptosis resulting in increased numbers of surviving cells. However, SP had no effect on either caspase-dependent or caspase-independent apoptosis of BMMCs as determined by cleaved caspase-3 and AIF, respectively. Therefore, maturation appears to be the major mechanism for increasing the number of effector MCs in the heart. There is a possibility of a contribution from recruited MC progenitor cells. Amadesi *et al*.^22^, reported that SP and the NK-1R induced bone marrow-derived progenitor cell recruitment in a mouse model of myocardial infarction. However, any recruited progenitor cells would become immature MCs and then be subject to maturation. Thus, assessment of maturation should be inclusive of contributions from progenitor cells. Also important to note is that MCs do not exist in the blood, therefore, MC migration to the heart is not a contributing factor.

SCF is essential for MC survival, proliferation and maturation^13,15–18^. We report that NK-1R antagonism prevented increased myocardial SCF in the SHR heart, identifying SCF as a likely mechanism by which the NK-1R regulates cardiac MC numbers. *In situ* hybridization identified endothelial cells, cardiomyocytes, as well as interstitial cells (likely fibroblasts) as the cellular sources of SCF. SP has been shown to induce SCF during bone marrow stroma formation, and in a reciprocal relationship, high binding affinity NK receptors can be induced by SCF^23^, thus, the up-regulation of SCF may also increase the availability of NK-1Rs. The reciprocal situation could also exist whereby the NK-1R could increase levels of c-kit, the receptor for SCF. However, using BMMCs as a surrogate for cardiac MCs, we found that SP did not increase c-kit.

Having identified the critical contribution of the NK-1R to MC maturation, we then determined whether the NK-1R induces MC activation and causes the release of MC factors (e.g. tryptase) that promote fibrosis. Initial *in vitro* conditioned media experiments showed that 3T3 fibroblasts increased the production of hydroxyproline in response to media from BMMCs, confirming the pro-fibrotic properties of MCs. However, fibroblasts did not synthesize additional hydoxyproline in response to conditioned media from BMMCs treated with SP, despite a small but statistically significant release of active tryptase by these BMMCs. Consequently, deletion of the NK-1R from BMMCs did not alter hydroxyproline synthesis by fibroblasts. This indicated that SP/NK-1R did not activate MCs sufficiently to cause a subsequent fibroblast response. To confirm these observations *in vivo*, we took advantage of the MC-deficient mouse (*Kit*^*w-sh/w-sh*^) whereby we could reconstitute these mice with MCs lacking the NK-1R. Firstly though, we confirmed that SP also contributed to cardiac fibrosis in mice by infusing *Tac1*^−/−^ mice with angiotensin II; cardiac fibrosis did not occur in these mice. Next, we confirmed that it was possible to reconstitute the hearts of *Kit*^*w-sh/w-sh*^ mice with MCs by injecting Q-dot labeled BMMCs. Other organs had previously been shown to reconstitute MC populations in the *Kit*^*w-sh/w-sh*^ mouse, however, the heart had not been examined^24^. Having confirmed successful reconstitution, we then reconstituted *Kit*^*w-sh/w-sh*^ mice with either wild type or *Nk-1r*^−/−^ BMMCs, thereby generating mice with either wild type MCs or MCs lacking the NK-1R. Angiotensin II infusion did not cause cardiac fibrosis in control *Kit*^*w-sh/w-sh*^ mice receiving saline instead of BMMCs. Conversely, *Kit*^*w-sh/w-sh*^ mice reconstituted with wild type BMMCs developed cardiac fibrosis. Together this confirms previous observations that MCs are involved in cardiac fibrosis^9^. Interestingly, *Kit*^*w-sh/w-sh*^ mice that received MCs deficient in the NK-1R also developed cardiac fibrosis. Thus, it appears that NK-1Rs on MCs are not required for MC activation in this setting. Others have shown that SP is able to cause degranulation of peritoneal, dural, skin, bladder and bone marrow MCs^8^. We also showed previously that high concentrations of SP (1000 nM) could activate cardiac MCs *in vitro*^7^. However, since this required high concentrations of SP, it raised the question as to whether this translates to the *in vivo* scenario. Our current study indicates otherwise, at least in this setting.

We wondered whether SP might indirectly cause MC activation via TNF-α. The rationale for this was two-fold: (1) SP and the NK-1R can regulate TNF-α levels in the heart^5^; and (2) crossing of TNF-α overexpressing mice with MC-deficient mice has demonstrated that TNF-α can activate MCs to cause fibrosis^19^, although whether this is a direct effect was unclear. We identified that cardiac MCs possess TNFRI, however, reconstitution of *Kit*^*w-sh/w-sh*^ mice with MCs deficient in TNFRI did not prevent fibrosis occurring in response to angiotensin II-infusion. Thus, TNF-α also does not appear to be a stimulus for direct activation of MCs in the fibrotic heart, at least in this model.

The results of our study add to an interesting debate. In an excellent review article by Shi *et al*.^25^, the authors suggest that SP is able to activate MCs via the NK-1R in cardiometabolic diseases. However, our results disagree with this, at least in the fibrotic heart. Newly emerging evidence suggests that SP-induced MC degranulation and inflammatory properties are not mediated through the NK-1R, but instead via MRGPRX2, a member of the Mas-related G protein coupled receptor (MRGPR) family^26–28^. Azimi and Lerner have pointed out that studies in the cardiometabolic disease field have used NK-1R antagonists instead of *Nk-1r*^−/−^ mice in showing that SP activation of cardiac and coronary MCs is NK-1R dependent^29^. Since NK-1R antagonists also have an inhibitory effect on Mrgprb2, the mouse orthologue of human MRGPRX2, this raises questions as to whether the actions of SP on MCs really are via the NK-1R in cardiometabolic diseases^26^. Our study clearly shows that fibrosis still occurs in the angiotensin II-infused mouse model with *Nk-1r*^−/−^ BMMC-reconstituted hearts, however, we cannot rule out the possibility that activation of Mrgprb2 on cardiac MCs by SP is involved in inducing cardiac fibrosis.

We also determined whether the same interactions between the NK-1R, SCF, and MC maturation existed in the angiotensin II mouse model. While we found that MCs were increased at 7 days in this model and tended to be reduced by SP deletion, there was a large variability in MC numbers. This may be a consequence of germ line deletion of SP versus blockade of its actions in the adult. To test this possibility, we took wild type mice and infused them with angiotensin II and treated them with the same NK-1R antagonist used in the SHR. In this case, NK-1R blockade prevented the increase in MC maturation, confirming the requirement of the NK-1R for MC maturation in the adult mouse, similar to the SHR. This increase is representative of MC maturation since avidin labels heparin sulfate, which is found in mature MCs. Interestingly though, unlike the SHR, angiotensin II mice did not exhibit an increase in SCF. When culturing BMMCs from mouse bone marrow, IL-3 is required in the culture media in addition to SCF to produce mature connective tissue MCs. It may be that the mouse uses IL-3 to promote maturation rather than a reliance on SCF as occurs in the rat and non-human primate^15–18,30^. However, SCF must make some contribution to the maintenance of MC numbers in mice, at least at baseline, since mice unable to respond to SCF due to c-kit mutations are MC-deficient, such as the mice used in this study.

Finally, we found no effect of NK-1R blockade on macrophage numbers in the SHR heart. However, we did observe that NK-1R blockade prevented the increase in myofibroblasts in the SHR heart. This may have occurred because by preventing the increase in MCs, NK-1R blockade prevents the known actions of MCs to increase myofibroblasts^9,10^. Thus, even though the NK-1R does not activate cardiac MCs, the equivalent effect may be achieved by NK-1R blockade simply by preventing the expansion of the mature MC population, hence, there are fewer cells available for activation. Fewer MCs means that there are also less proteases available to exert downstream effects, as demonstrated by tryptase levels in the SHR receiving NK-1R blockade. This has real functional consequences given the pro-fibrotic effects of tryptase on cardiac fibroblast phenotype and function as well as its contribution to fibrosis in the hypertensive heart^10^. Alternatively, SP activation of the NK-1R may directly regulate myofibroblast number. Kumaran *et al*.^31^ had previously reported that SP caused cardiac fibroblast proliferation. It is important to keep in mind, however, that while SP induces proliferation it does not appear to directly stimulate cardiac fibroblasts to produce excess collagen, at least *in vitro*^3,4,31^.

**Figure 6:**
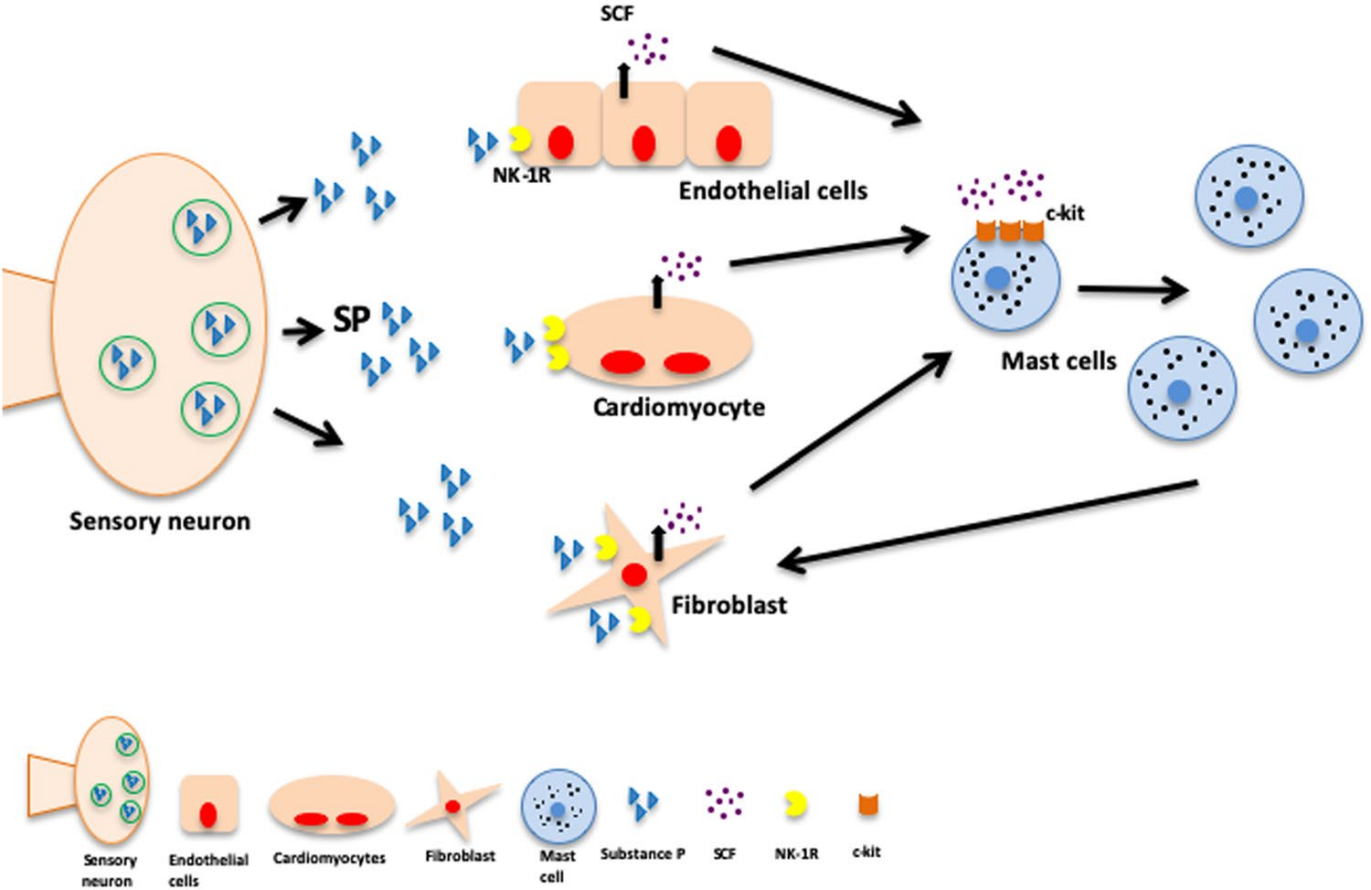
Schematic depiction of SP and the NK-1R regulation of cardiac MCs and fibrosis. SP activation of the NK-1R causes an increase in cardiac MC density in the heart by increasing SCF production by endothelial cells, cardiomyocytes, and fibroblasts. While MCs are known to cause fibrosis, the NK-1R is not required for the activation of cardiac MCs. SP also acts to increase the number of myofibroblasts, however, it is not clear whether this is a direct effect on myofibroblast proliferation or a by-product of increasing the number of mature MCs that then act to increase myofibroblast numbers.

In summary, we sought to determine the extent to which cardiac MCs mediated the pro-fibrotic actions of the NK-1R. We found that SP/NK-1R is essential to increase the number of mature MCs in the hypertensive heart and does this by increasing the production of SCF. However, the NK-1R is not required for activation of cardiac MCs *in vivo* (Fig. 6). This study establishes a neuro-immune component to the development of cardiac fibrosis.

## Supplementary information

Supplementary Information

## Data Availability

The datasets generated during and/or analysed during the current study are available from the corresponding author on reasonable request.
