# Supplementary material for: Regulation of Cardiac Mast Cell Maturation and Function by the Neurokinin-1 Receptor in the Fibrotic Heart
Source: Sci Rep. 2019 Jul 29;9:11004. doi: 10.1038/s41598-019-47369-0 (PMC6662794; doi:10.1038/s41598-019-47369-0)
Supplement: Supplementary file 1 — Supplementary Information [file 41598_2019_47369_MOESM1_ESM.docx]

**SUPPLEMENTARY INFORMATION**

**Regulation of Cardiac Mast Cell Maturation and Function by the Neurokinin-1 Receptor in the Fibrotic Heart**

^1,2^Alexander Widiapradja, ^3^Edward J. Manteufel, ^3^Heather M. Dehlin, ^4^James Pena, ^4^Paul H. Goldspink, ^3^Amit Sharma, ^3^Lauren L. Kolb ^3^John D. Imig, ^5^Joseph S. Janicki, ^6^Bao Lu, ^1,2^Scott P. Levick

^1^Kolling Institute of Medical Research, Royal North Shore Hospital, St Leonards, NSW, 2065

^2^Faculty of Medicine and Health, The University of Sydney, Camperdown, NSW, 2006

^3^Department of Pharmacology and Toxicology, Medical College of Wisconsin, Milwaukee, WI, 53226

^4^Department of Physiology, Medical College of Wisconsin, Milwaukee, WI, 53226

^5^Cell Biology and Anatomy, University of South Carolina School of Medicine, SC, 29208

^6^Division of Respiratory Diseases, Boston Children’s Hospital, Harvard Medical School, Boston, MA, 02115

**Corresponding Author:**

Scott P. Levick, Ph.D., FAHA

Kolling Institute of Medical Research

The University of Sydney

St Leonards, New South Wales, 2065

Telephone +61 2 9926 4911; email [scott.levick@sydney.edu.au](mailto:scott.levick@sydney.edu.au)

**Methods**

*Osmotic mini-pump implantation*

Mice were anesthetized using inhaled isoflurane not exceeding 2.5%. Briefly, a laparotomy was performed and the mini-pump (Alzet) placed in the abdominal cavity. The abdominal musculature was closed using 5-0 chromic gut sutures, whilst the skin wound was closed with surgical staples. Analgesia was achieved using buprenorphine (0.05 mg/kg).

*Bone marrow-derived mast cells (BMMC)*

Bone marrow cells were extracted from the tibia and femur bones of 6-7 week old male wild type, *Nk-1r^-/-^*, or *TnfrI^-/-^* mice and centrifuged at 800 rpm for 8 minutes in Dulbecco’s Modified Eagle’s Medium (DMEM) supplemented with penicillin/streptomycin and 10% FBS. The pellet was then resuspended in medium containing 10 ng/mL of rmIL-3 and 50 ng/mL of rmSCF (Peprotech). Non-adherent cells were harvested 1-2 times per week for at least 6 weeks to obtain a BMMC culture at least 90% pure. To confirm purity of cultures, cells were labeled with anti-FcεRI (IgE receptor) before placing on a slide for microscopic analysis. For *in vitro* experiments, BMMCs were treated with SP at increasing concentrations (0, 100, 300, and 1000 nM) for 24 hrs. All experiments contained SCF (50 ng/mL) and IL-3 (10 ng/mL) during the treatment period. We believe that BMMCs serve as an acceptable model of cardiac mast cells (MCs) for the purposes of this study because, while there are almost certainly phenotypic differences, both BMMCs and cardiac MCs are tryptase^+^/chymase^+^, thus, both meet the classification of connective tissue type MCs ^1^. Further, flow cytometry determined that almost all BMMCs and cardiac MCs possess the NK-1R (Figure 2C and Figure 4C and D). BMMCs allowed us to generate the number of cells required, but from fewer animals.

*BMMC reconstitution of Kit^w-sh/w-sh^* *mice*

Firstly, to determine the feasibility of reconstituting the hearts of *Kit^w-sh/w-sh^* mice with BMMCs, wild type BMMCs were labeled in culture with fluorescent quantum dots (Q dots) using the Qtracker Cell Labeling Kit (Life Technologies), according to the manufacturer’s protocol. Q dots are taken up by the cell and then retained in the cell allowing the tracking of BMMCs to confirm their reconstitution in the left ventricle (LV). Following Q dot labeling, BMMCs were reconstituted into *Kit^w-sh/w-sh^* mice using methods modified from Kitamura et al., ^2^ and Wolters et al., ^3^. Briefly, 1x10^7^ BMMCs/200 µL of saline were injected via the tail vein into *Kit^w-sh/w-sh^* mice. Six weeks post injection, the mice were euthanized, the LV processed for histology and the presence of Q dot-containing MCs viewed via a fluorescent microscope. Subsequently, to determine the contribution of MC-specific NK-1R and TNFRI to cardiac fibrosis, *Kit^w-sh/w-sh^* mice were reconstituted with either wild type BMMCs, *Nk-1r^-/-^* BMMCs, or *TnfrI^-/-^* BMMCs and infused with angiotensin II. Saline tail vein injections in *Kit^w-sh/w-sh^* mice not receiving BMMCs served as controls for the BMMC injection.

*Histology*

For MC identification in rat LV, 5 μm sections underwent dehydration before staining with toluidine blue in 1% NaCl for 30 seconds and brief rinsing with water. Cardiac MCs stained with toluidine blue (purple) were counted in each LV section and normalized to LV section area. Toluidine blue is not effective for staining murine MCs, therefore, MCs in mouse LVs were labeled with avidin conjugated to a 488 fluorochrome. Avidin specifically labels MC granules ^4^.

To differentiate between mature and immature MCs in rats, LV tissue at 5 μm thickness underwent dehydration before staining with 0.5% alcian blue (in pH 1 PBS) for 45 minutes. The LV sections were then washed with PBS (pH 1) before staining with 0.5% safranin (in pH 1 PBS) for a further 45 minutes. The LV sections were then washed with PBS (pH 1), rehydrated, and cover slipped. For each LV section, the percentage of immature (blue) and mature (violet-orange/orange color) MCs were determined from the total number of MCs (i.e. blue stained MCs + violet-orange/orange MCs) as we have described previously ^5^. The staining of MCs with alcian-blue or safranin relates to proteoglycan synthesis during the maturation process. Immature MC cytoplasmic granules contain the weakly sulfated polysaccharide, chondroitin sulfate, which absorbs alcian-blue. As heparin proteoglycan synthesis increases with maturation, this results in the ability to absorb the safranin stain resulting in a violet-orange and finally orange color as MCs fully mature.

For collagen staining, slides containing formalin fixed mouse LV sections at 5 μm thickness underwent dehydration before incubation in phosphomolybdic acid (0.2%) and then staining with picrosirius red (0.1% Sirius Red F3BA in picric acid). All slides underwent rehydration before being mounted and coverslipped with DEPEX. Collagen volume fraction was determined as previously described ^6,7^. Ten images per LV section were acquired and analyzed with Image J software. Perivascular areas were excluded from the collagen analysis.

*In situ hybridization*

The protocol used was adapted and modified from Christensen et al ^8^. Briefly, slides containing LV tissues were rehydrated in xylene and ethanol before being fixed in 4% paraformaldehyde. The tissues were permeabilized with 10 μg/mL of proteinase K for 20 minutes and incubated with the complementary (antisense) RNA probe overnight at 65°C in a sealed humidified chamber. Once the excess probe was removed the tissues were blocked in blocking buffer containing 10% goat serum and 2% BSA for 1 hour. Alkaline phosphatase–conjugated anti-digoxigenin IgG (Roche) was added to the blocking solution and incubated overnight at 4°C. The next day, the tissues were incubated in 40 mg/ml NBT and 20 mg/ml 5-bromo-4-chloro-3-indolylphosphate (Roche) at 37°C overnight for color development. The reaction was stopped by immersing the tissues in PBS, and followed by mounting with DEPEX. A sense RNA probe was used as a negative control.

*Conditioned media experiments*

Mouse BMMCs were treated with SP (0, 300, and 1000 nM) for 24 hours. The conditioned media was collected and transferred to NIH/3T3 fibroblasts (ATCC® CRL-1658™) cultured in 6 well plates at 5x10^5^ cells/well for an additional 24 hours before the media was collected for hydroxyproline analysis.

*Genotyping*

To confirm deletion of the NK-1R in BMMCs, wild type and *Nk-1r*^-/-^ BMMCs were generated as described above and DNA extracted using a Qiagen DNeasy kit (Qiagen) according to the manufacturer’s protocol. The PCR reaction was performed using the Tera PCR polymerase kit (Takara) and the products run on a 1.5% agarose gel at 100V for 1 hour in 1x TAE buffer and stained with EtBr. The primers used were mouse *Nk-1r,* forward, 5’-GTG CAA CCT ACC TGG CAA AT-3’, reverse 5’-TGT GGA CTG CGT AGG TGA AG-3’ with the product size of 211 base pairs (bp).

*Immunobloting*

LV tissue (100 mg) from WKY, SHR, and SHR treated with L732138 was homogenized in ice cold PBS and briefly sonicated on ice. Protein lysate and Laemmli buffer with β-mercaptoethanol (1:1) was boiled for 5 minutes prior to loading on a 15-4% precast gradient gel (Bio-Rad). Samples were transferred to a flourochrome optimized PVDF membrane (Immobilon-FL, Millipore) and irradiance was detected using flourochrome-conjugated secondary antibodies and an Odyssey imaging system (Li-Cor Biosciences). Primary antibodies used were rabbit anti-mast cell tryptase (sc-33676 Santa Cruz) at a 1:100 concentration and mouse anti-GAPDH (Calbiochem) at a 1:1000 concentration.

Membranes containing protein lysates from mouse BMMCs that had been separated in 4-15% precast gradient gel (Bio-Rad) were incubated with primary antibodies against cleaved caspase 3 (1:1000, Cell Signaling), apoptosis inducing factor (AIF, 1:1000, Cell Signaling), and GAPDH (1:1000, MerckMillipore). The primary-secondary antibody reaction was visualized using Odyssey imaging system (Li-Cor Biosciences). Images of full blots are provided in Figure S7.

*Immunofluroescence*

To assess MC proliferation, SHR LV sections at 5 μm thickness were labelled with anti-Ki67 (1:100) following blocking for non-specific binding. Ki67 labelling was detected with 488-conjugated goat anti-rabbit secondary antibody (1:100, Life Technologies). MCs were labeled with alcian blue.

For macrophage and myofibroblast labeling, formalin fixed rat LV sections of 5 μm thickness underwent dehydration before incubation in pH 9 buffer for 30 min in a pressure cooker for antigen retrieval. The sections were labelled with anti-CD68 (1:100, Life Technologies) to identify macrophages and anti-α-smooth muscle actin (1:50, Abcam) to identify myofibroblasts following blocking for non-specific binding. CD68 labelling was visualized using 488-goat anti-mouse secondary antibody (1:100, LifeTechnologies), while α-smooth muscle actin was visualized using 568-goat anti-rabbit secondary antibody (1:100, LifeTechnologies). All sections were cover slipped with prolong-Diamond containing DAPI (LifeTechnologies) and visualized under a fluorescence microscope. The number of macrophages was determined by counting the total number of CD68^+^ cells in each LV section. The number of myofibroblasts was determined by counting the total number of interstitial anti-α-smooth muscle actin^+^ cells in each LV section. α-smooth muscle actin^+^ cells associated with blood vessels were excluded from the analysis.

*Biochemical assay*

Commercially available ELISA kits were utilized to determine the protein levels of SCF (Abcam) from rat and mouse LV, and the c-kit receptor (Abcam) from mouse BMMCs. BMMC tryptase activity was determined using the Mast Cell Activity Assay (MerckMillipore). All samples were run in duplicate according to manufactures’ protocols.

*Hydroxyproline assay*

100 µL of cell culture media was incubated with 100 µl of 6N HCl and hydrolyzed at 107 ºC for 18 hours. The samples were dried and reconstituted with 500 μL of dH2O. They were then oxidized with 250 μL of chloramine T reagent and developed with 250 μL of Ehrlich’s reagent. Absorbance was read at 550 nm and hydroxyproline values determined from a hydroxyproline standard curve.

*BMMC proliferation assay*

BMMCs were cultured in 96 well plates at 1x10^4^ cells/well for 24 hours before undergoing SP treatment (0, 100, 300, 1000 nM) for an additional 24 hours. Cell proliferation was determined using the CyQuant NF Proliferation assay Kit (Life Technologies) according to the manufacturer’s protocol. The assay was performed in duplicate and read using a fluorescence microplate reader at 485 nm wavelength.

*Flow cytometry*

Mice were euthanized and the hearts collected in ice cold saline. The LV and septum were isolated from the RV. The LVs were subsequently digested (DMEM containing 1 mg/mL collagenase, 0.1 mg/mL DNAse I) at 37°C for 30 minutes and passed through a 40 μm cell strainer. Cells were then incubated with standard erythrocyte lysis buffer on ice and separated from debris by Percoll gradient (GE Healthcare) centrifugation. Cells were surface stained and fixed in fixing buffer before being run through a LSR II FACS system. The data obtained were analyzed with FlowJo. The antibodies used were FceR-FITC (1:100, eBioscience), NK-1R-PE (1:100, NovusBiologicals), and TNFRI-PE (1:100, eBioscience). Gating strategies are shown in Figures S1 and S2, as well as Figure S5.

*Statistical analysis*

All data were expressed as mean ± standard deviation (SD) or standard error of the mean (SEM) as appropriate. Multiple group data comparisons were made by one-way ANOVA with Tukey post-test, except when two variables are compared in which case a two-way ANOVA was used. Student t-test was used when there were only two groups compared. P ≤ 0.05 was deemed significant.

**Figure S1.** Gating strategy for identifying FcεR1^+^/ NK1R^+^ BMMCs by flow cytometry. Gating for FcεR1^+^ mast cells **(B)** was determined from baseline using unstained bone marrow derived mast cells (**A**). This population was then analyzed for FcεR1^+^/ NK1R^+^ expression (**C**).

Fc𝛆R1^+^/NK1R^+^


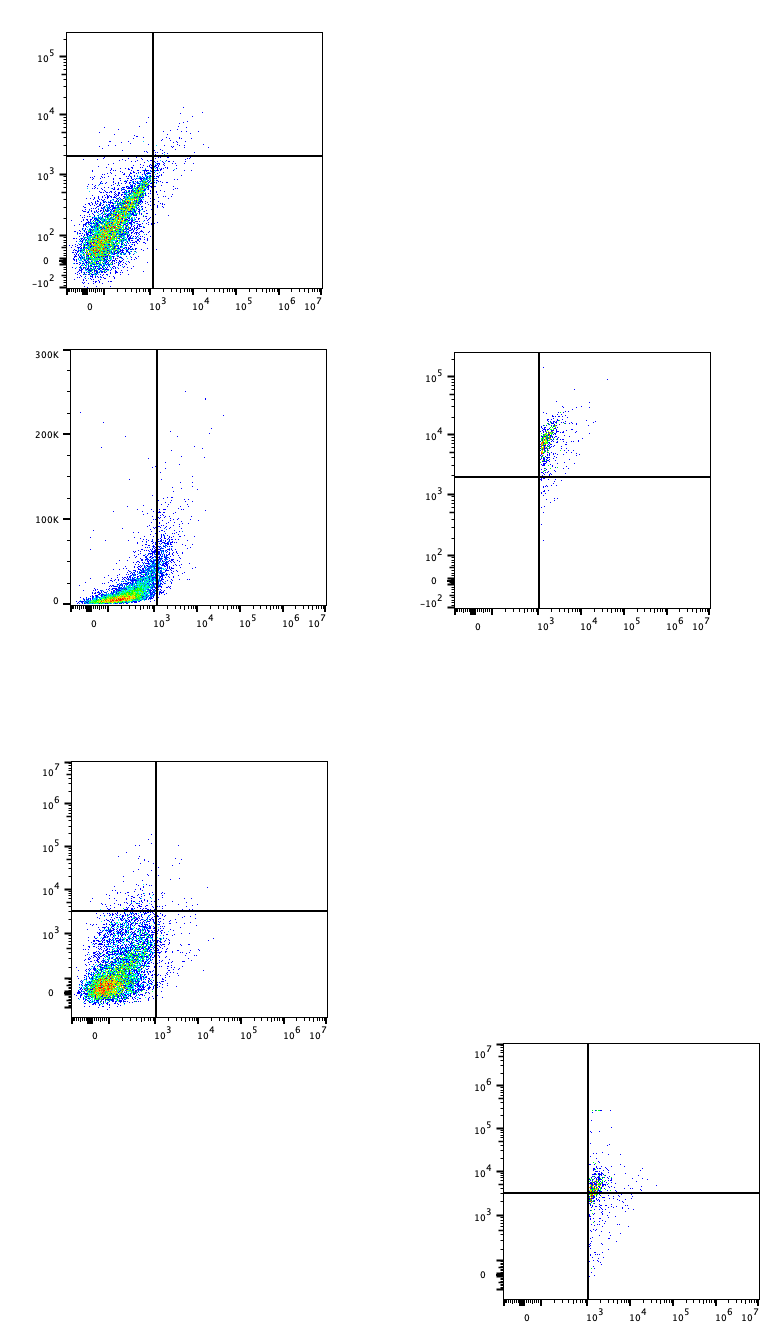


Unstained

Fc𝛆R1^+^

NK1R^+^


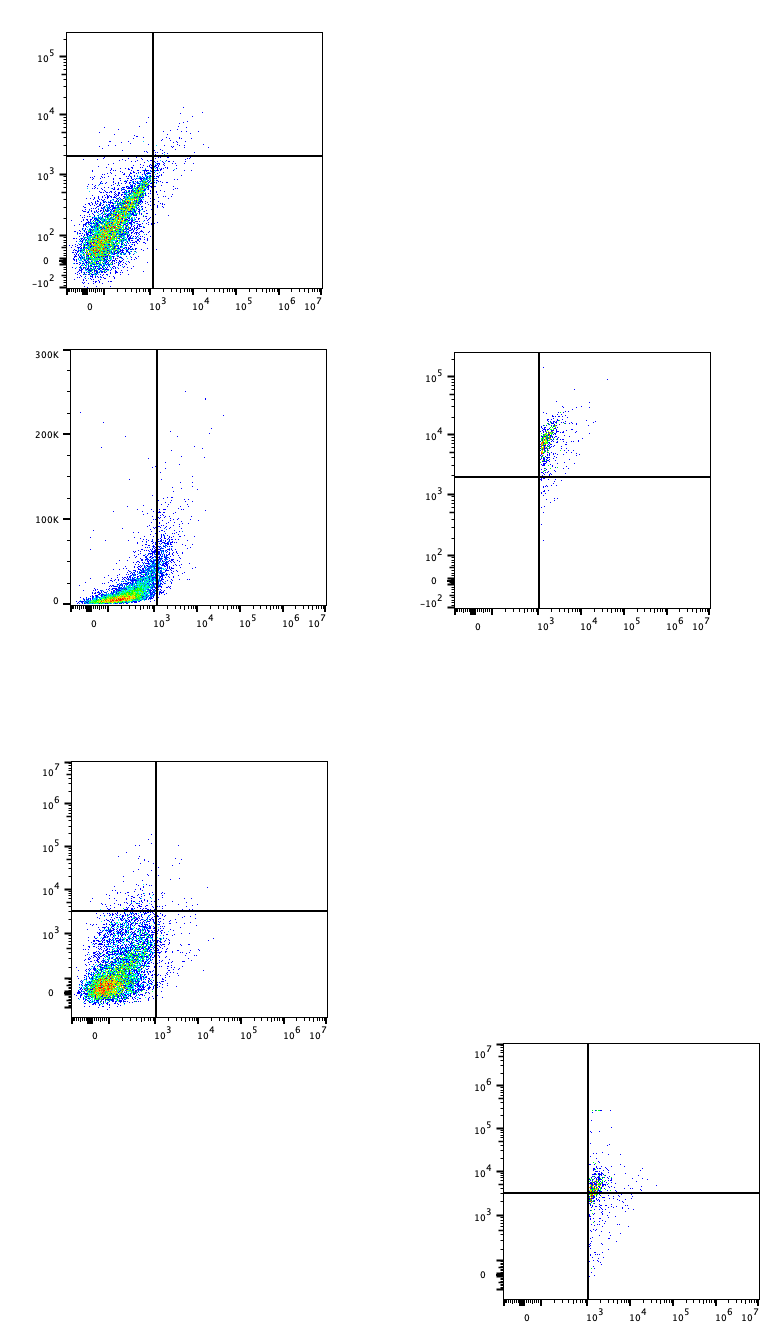

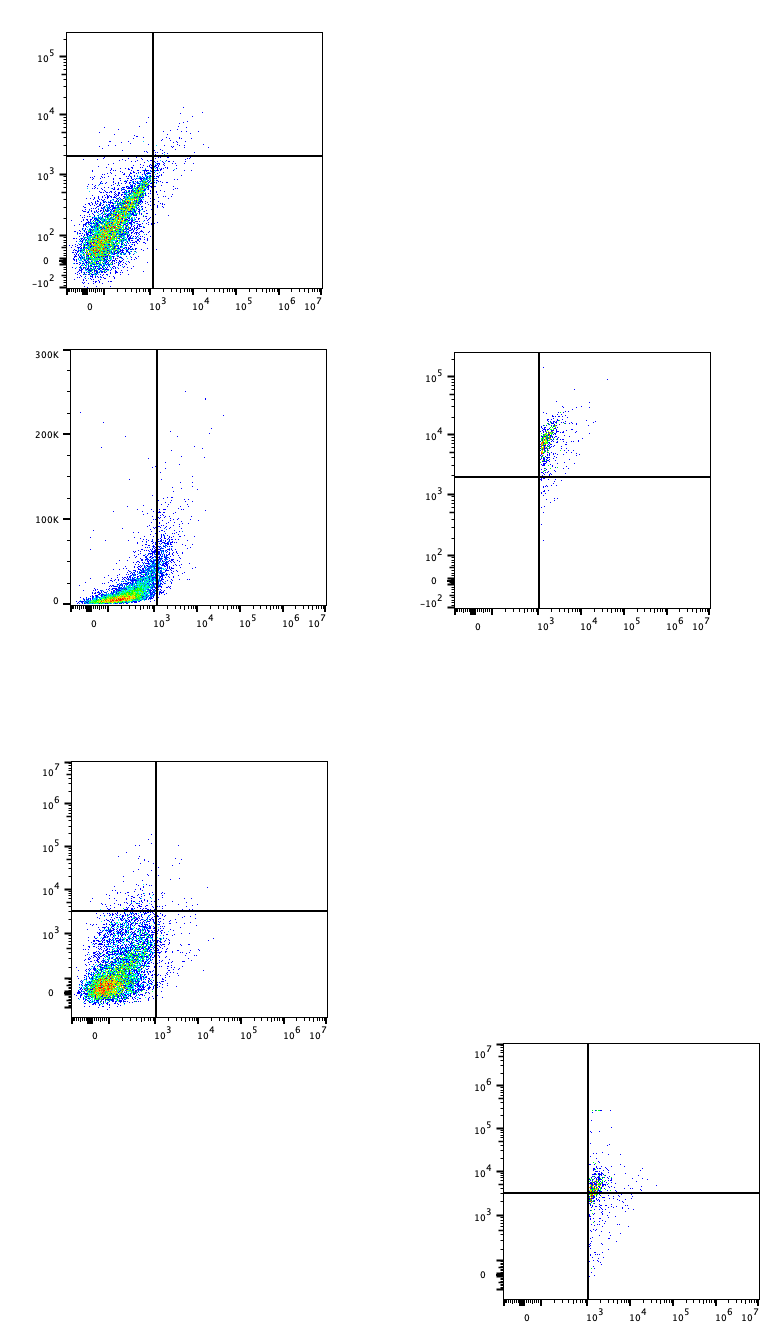


NK1R^+^

SSC-A

**A**

**B**

**C**

**Figure S2.** **Gating strategy for identifying FcεR1^+^/ NK1R^+^ cardiac mast cells by flow cytometry. (A)** The unstained isolated cardiac leukocyte population was used to set gates for FcεR1 (mast cells) and the NK-1R; **(B)** Following antibody labelling, cells shifted to the right of the gate were considered to be FcεR1^+^ (right hand panel). The top right quadrant of **(C)** then shows cells that were FcεR1^+^/ NK1R^+^, indicating mast cells that expressed the NK-1R.











50 μm

**Figure S3.** Representative images of successful reconstitution of *Kit^wsh/wsh^* mouse hearts with BMMCs labelled with Q dots (400x mag.). Arrows indicate mast cells.

**Figure S4.** MC number in LV sections from successful mast cell reconstitution of *Kit^wsh/wsh^*+saline+Ang II, *Kit^wsh/wsh^*+WT BMMC+Ang II, *Kit^wsh/wsh^*+*Nk-1r^-/-^* BMMC+Ang II, and *Kit^wsh/wsh^*+*Tnfr1^-/-^* BMMC+Ang II mice.


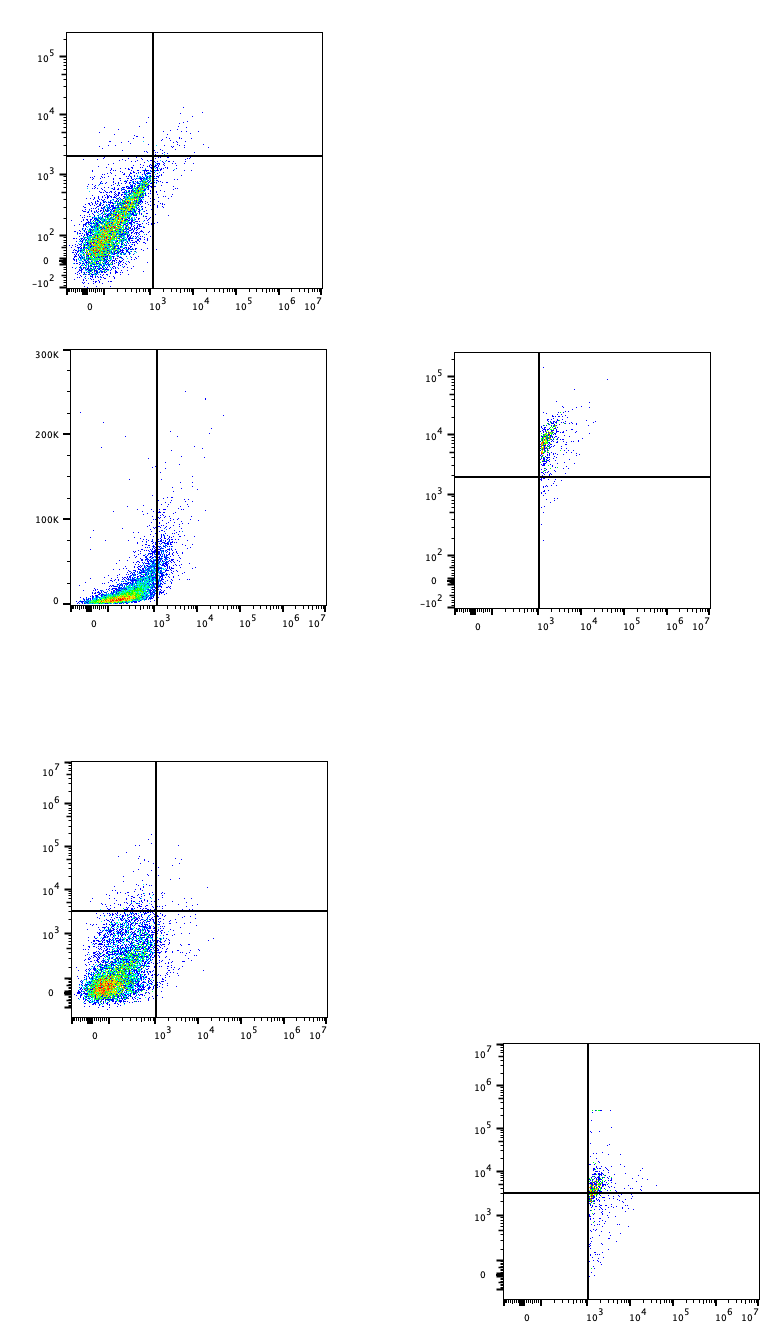


Unstained


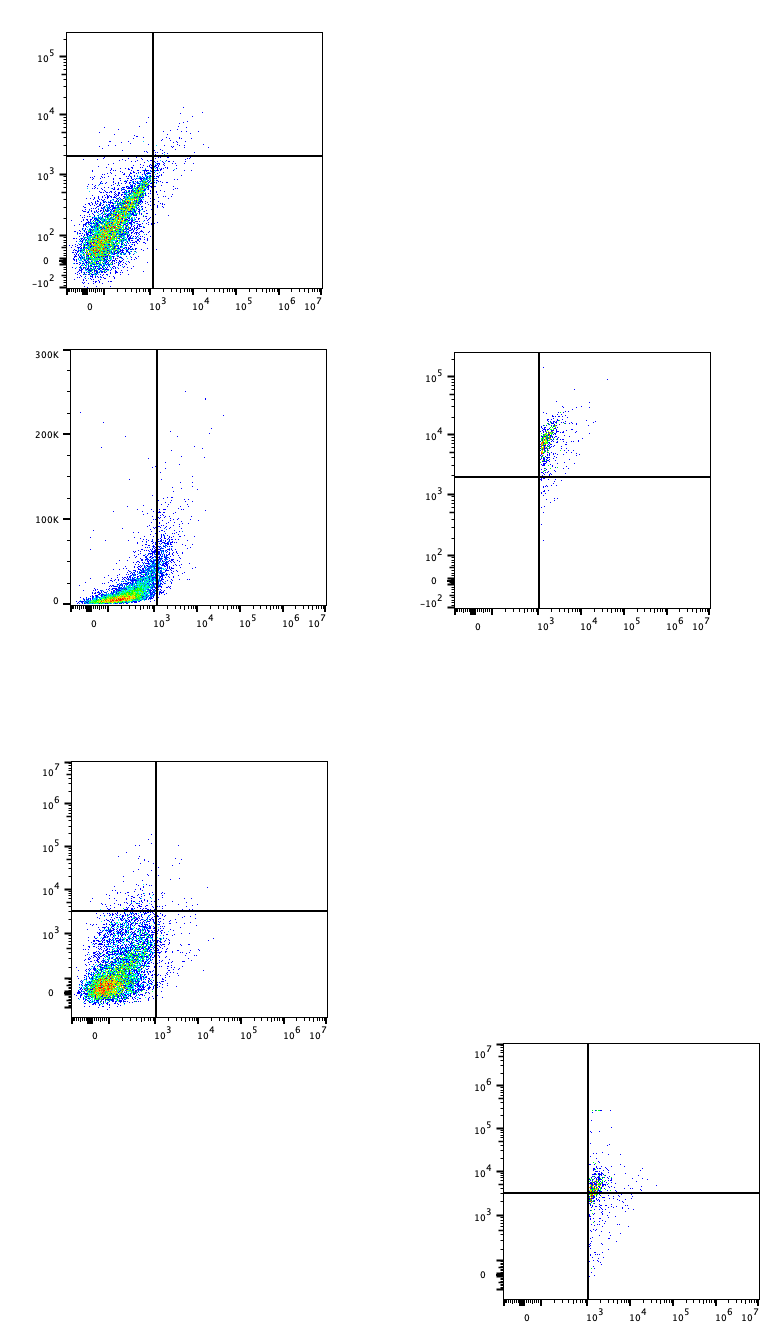


SSC-A


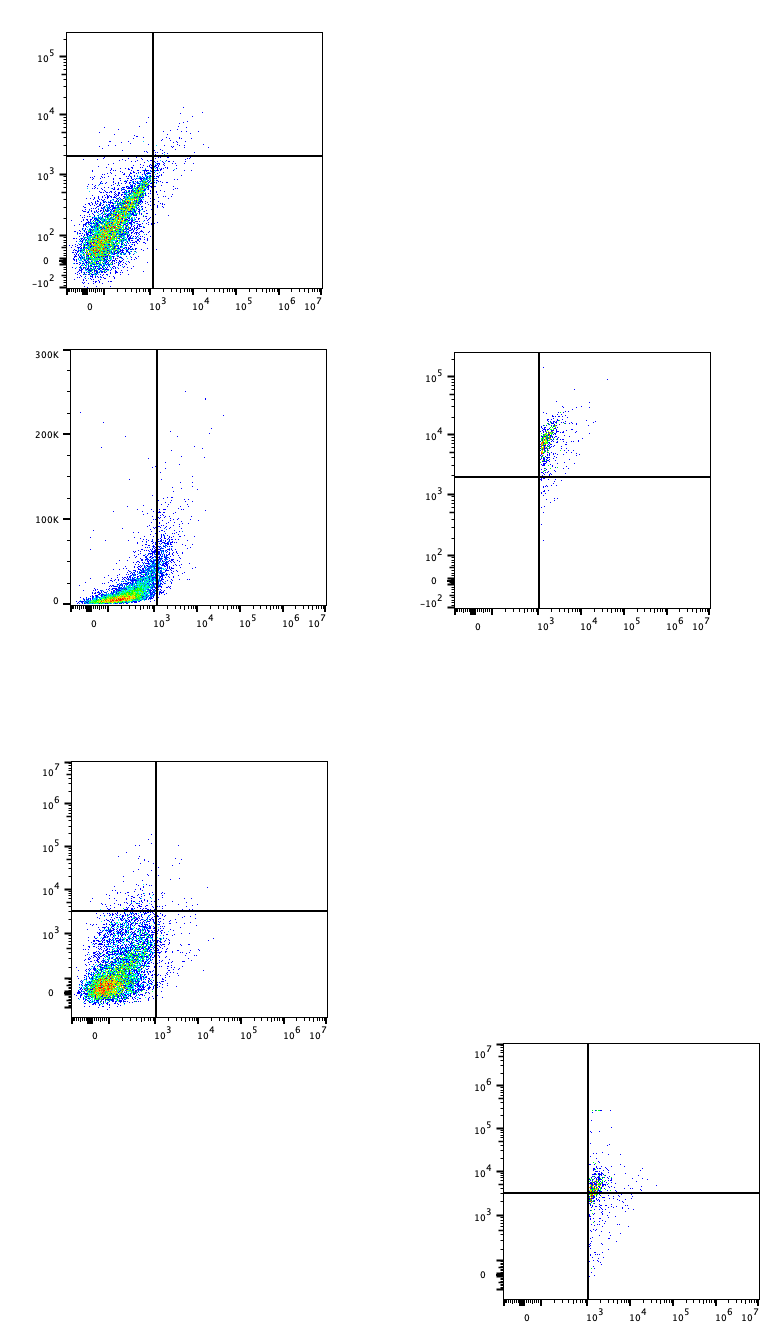


Fc𝛆R1^+^

TNFR1^+^

TNFR1^+^

**A**

**B**

**C**

Fc𝛆R1^+^/TNFR1^+^

**Figure S5.** **Gating strategy for identifying FcεR1^+^/ TNFRI^+^ cardiac mast cells by flow cytometry. (A)** The unstained isolated cardiac leukocyte population was used to set gates for FcεR1 (mast cells) and TNFRI; **(B)** Following antibody labelling, cells shifted to the right of the gate were considered to be FcεR1^+^ (right hand panel). The top right quadrant of **(C)** then shows cells that were FcεR1^+^/TNFRI^+^, indicating mast cells that expressed the TNFRI.

**B**

**A**

**C**

**Figure S6. (A)** Number of mature MCs (avidin^+^) in LV sections from 7-day WT Saline, WT Ang II, *Tac1^-/-^* Saline, and *Tac1^-/-^* Ang II mice; **(B)** Number of mature MCs (avidin^+^) in LV sections from 1-day WT Saline, WT Ang II, and WT Ang II mice treated with L732138 (5 mg/kg/d); **(C)** cardiac stem cell factor (SCF) levels for 1-day WT Saline, WT Ang II, and WT Ang II mice treated with L732138. Data are mean ± SD (A and B) and SEM (C), *=p<0.05 vs Saline.

**
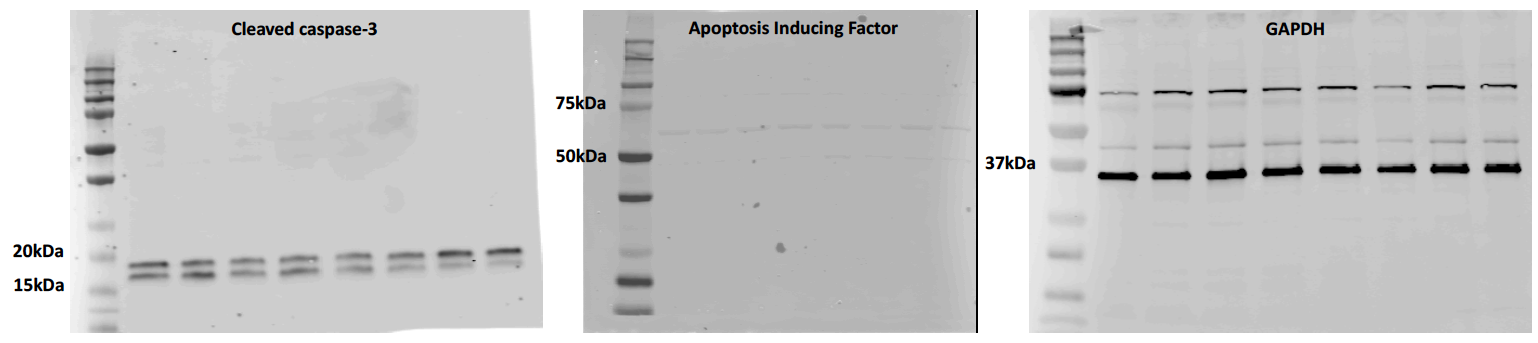
Figure S7.** Images of full-length western blots for cleaved caspase-3, apoptosis inducing factor, and GAPDH that appear in cropped form in Figure 2.

**References**

1 Bischoff, S. C. Role of mast cells in allergic and non-allergic immune responses: comparison of human and murine data. *Nat Rev Immunol* **7**, 93-104 (2007).

2 Kitamura, Y., Go, S. & Hatanaka, K. Decrease of mast cells in W/Wv mice and their increase by bone marrow transplantation. *Blood* **52**, 447-452 (1978).

3 Wolters, P. J. *et al.* Tissue-selective mast cell reconstitution and differential lung gene expression in mast cell-deficient Kit(W-sh)/Kit(W-sh) sash mice. *Clinical and experimental allergy : journal of the British Society for Allergy and Clinical Immunology* **35**, 82-88 (2005).

4 Tharp, M. D., Seelig, L. L., Jr., Tigelaar, R. E. & Bergstresser, P. R. Conjugated avidin binds to mast cell granules. *J Histochem Cytochem* **33**, 27-32 (1985).

5 Li, J. *et al.* Stem cell factor is responsible for the rapid response in mature mast cell density in the acutely stressed heart. *J Mol Cell Cardiol* **53**, 469-474 (2012).

6 Dehlin, H. M., Manteufel, E. J., Monroe, A. L., Reimer, M. H., Jr. & Levick, S. P. Substance P acting via the neurokinin-1 receptor regulates adverse myocardial remodeling in a rat model of hypertension. *Int J Cardiol* **168**, 4643-4651 (2013).

7 Melendez, G. C., Manteufel, E. J., Dehlin, H. M., Register, T. C. & Levick, S. P. Non-human primate and rat cardiac fibroblasts show similar extracellular matrix-related and cellular adhesion gene responses to substance P. *Heart Lung Circ* **24**, 395-403 (2015).

8 Christiansen, J. H. *et al.* Murine Wnt-11 and Wnt-12 have temporally and spatially restricted expression patterns during embryonic development. *Mech Dev* **51**, 341-350 (1995).
